# Retraction Note: The POU2F1/miR-4490/USP22 axis regulates cell proliferation and metastasis in gastric cancer

**DOI:** 10.1007/s13402-022-00768-4

**Published:** 2022-12-29

**Authors:** Yizhi Xiao, Side Liu, Jiaying Li, Weiyu Dai, Weimei Tang, Li Xiang, Wenjing Zhang, Jianjiao Lin, Jing Wang, Xiaosheng Wu, Guangnan Liu, Yuyang Liu, Yaying Chen, Huiqiong Zhu, Yusi Wang, Zhizhao Lin, Qiong Yang, Tianming Chen, Yong Sun, Aimin Li, Jing Xiong, Jide Wang

**Affiliations:** 1 Guangdong Provincial Key Laboratory of Gastroenterology, Department ofGastroenterology, Department of Digestive Medicine, Nanfang Hospital, Southern Medical University, Guangzhou, 510515 Guangdong Province China; 2 Department of Gastroenterology, Longgang District People’s Hospital, Shenzhen, 518172 China; 3 Department of Medical Oncology, the First people’s Hospital of Yunnan Province, Medical School of KunmingUniversity of Science and Technology, Kunming, 650032 China; 4 The Second School of Clinical Medicine, Southern MedicalUniversity, Guangzhou, Guangdong 510515 People’s Republic of China; 5 Department of Gastroenterology, The third affiliated hospital of Guangzhou medical university, Guangzhou, 510150 China; 6 The Second Affiliated Hospital of University of South China, Hengyang, 421001 China

**Retraction Note: Cellular Oncology (2020) 43:1017–1033**

10.1007/s13402-020-00553-1

The Editors-in-chief have retracted this article because after publication concerns have been raised regarding a number of figures, specifically:

- Figure 3a: the 24h panels for m-NC and i-NC appear to be identical.
- Figure 4f: the AGS and BGC-823 panels for miR-4490/USP22 appear to partially overlap.

The Editors-in-Chief therefore consider the data reported in this the article to be unreliable.

Jide Wang does not agree to this retraction. The editor was not able to obtain a current email address for Tianming Chen. None of the other authors have responded to any correspondence regarding this retraction.

